# The Fungi–Bacteria Interaction Mechanism of Microbial Consortium During Efficient Lignin Degradation Based on Metabolomics Analysis

**DOI:** 10.3390/molecules30030508

**Published:** 2025-01-23

**Authors:** Wen Zhang, Yilei Wen, Zhequan Wang, Chenyang Diao, Zhiwei Liu

**Affiliations:** 1 Key Laboratory of Pollution Exposure and Health Intervention of Zhejiang Province, College of Biology and Environmental Engineering, Zhejiang Shuren University, Hangzhou 310015, China; yilei0430@126.com (Y.W.); 13757575939@163.com (Z.W.); x_yang24@163.com (C.D.); 2 College of Environment and Chemical Engineering, Yanshan University, Qinhuangdao 066004, China; zwliu@ysu.edu.cn

**Keywords:** lignin degradation, microbial consortium, metabolomics analysis, interaction mechanism

## Abstract

Microbial consortium degradation technology can improve the degradation efficiency and adaptability through fungi–bacteria synergism, but the mechanism of the fungi–bacteria interaction is still unclear, making it difficult to optimize the degradation process. The microbial consortium J-6, with high lignin degradation efficiency and strong environmental adaptability, was obtained in our previous research. In this study, the fungi–bacteria interacting mechanism of the microbial consortium J-6 was inferred based on metabolomics technology. The results showed that the positive interaction between fungi and bacteria could improve the efficiency of lignin degradation. The metabolites released by fungi, especially betanidin and ergosterol, had an impact on bacterial metabolism, promoted the degradation of macromolecules, and significantly increased the lignin degradation efficiency. Metabolites released by bacteria, especially L-phenylalanine and taurine, played a key role in fungal metabolism, leading to more complete degradation. The interaction mechanism of chemical currencies exchange between fungi and bacteria during lignin degradation obtained in this study can provide theoretical guidance for microbial consortium degradation technology.

## 1. Introduction

The lignocellulosic biomass has wide sources on Earth. The annual production of lignocellulose can reach 170 billion tons; however, only 3% of lignocellulose can be effectively utilized in the circular bioeconomy [1]. The effective utilization of lignocellulosic biomass can not only reduce environmental pollution and carbon emissions but also produce bioethanol and other fuels to relieve fossil energy shortages [2]. Lignin, as a major component of plant cell walls, not only provides strength and resistance to lignocellulosic biomass but also protects carbohydrates from enzyme digestion. The degradation of lignin is a key step in the utilization of lignocellulosic resources [3]. However, lignin, as a macromolecular aromatic polymer, is difficult to degrade due to its complex structure [4]. At present, the degradation process of lignin has been a research hotspot in the field of global carbon cycle and biomass resource utilization.

Lignin can be degraded by many methods, such as biodegradation, catalytic degradation [5,6], solvent degradation [7], and so on. By comparison, microbial degradation has the advantages of low energy consumption, low cost, environmental friendliness, and safe operation. However, single fungi always grow and degrade slowly, while bacterial degradation has the disadvantages of incomplete degradation and low degradation efficiency [8]. As a result, the research on the lignin degradation system of fungi–bacteria mixed microorganisms has attracted a lot of attention. Iimura et al. [9] found five species of bacteria of the genus *Methylobacterium* that coexisted with white-rot fungi from decayed wood, and their research showed that the lignin degradation rate was increased by the fungi–bacteria collaboration. Zhong et al. [10] also revealed the effectiveness of fungi–bacteria synergistic pretreatment for the lignin degradation process. In our preliminary research, four efficient lignin-degrading microbial consortia were screened, among which the lignin degradation efficiency by microbial consortium J-6 could reach 54% in 2 d. Moreover, microbial consortium J-6 could effectively and selectively degrade lignin in the biomass degradation system (selectivity value = 3.59), which proved that fungi and bacteria could effectively increase the degradation efficiency of lignin in lignin degradation systems through positive interactions [11].

The degradation mechanism of lignin by the single strain has been extensively investigated [12]. However, research on the degradation mechanism of the microbial consortium to degrade lignin is insufficient. Although there were reports on the possible ways of fungi–bacteria interactions in the microbial consortium during lignin degradation, the mechanisms of fungi–bacteria interaction during lignin degradation still remain unclear. For example, Wilhelm et al. [13] reported that the fungi carried out the primary degradation of lignin, and the bacteria continued to metabolize and synthesize their cells subsequently. In the study of lignin decomposition processes in soil, Lacerda-Junior et al. [14] found that key bacterial (Bordetella and Streptomyces) and fungal (Aspergillus) members may play synergistic eco-physiological roles in the decomposition of lignin-derived compounds through the carriage of most of the genes encoding auxiliary activity (AA) family of lignin-modifying enzymes. It is worth noting that these studies lacked exploration of interacting substances in microbial consortiums, resulting in unclear mechanisms of interaction.

Currently, the mechanism of interactions among microorganisms in the microbial consortium has attracted extensive attention. For example, substances released by microorganisms may become signal transducers that activate or inhibit gene expression or biological activity, thereby altering the behavior and growth of other microorganisms. Amin et al. [15] found that the indoleacetic acid secreted by *sulfitobacter* species could stimulate the division of diatom cells and promote the growth of algae. Song et al. [16] used metabolomics analysis to study the interacting substance between chlorella vulgaris and *microcystis*. Bruger et al. [17]’s research suggests that bacteria produce and release small hormone-like signaling molecules called autoinducers (AI) into the environment and that adjacent microbial cells detect and respond to these molecules to change gene expression and coordinate the behavior of the entire community. Shibl et al. [18] identified a key chemical currency in the regulation of microbial interactions in different ecosystems.

Meanwhile, the use of metabolomics technology has made positive progress in terms of the mechanism of microbial lignin degradation in recent years [19,20]. Metabolomics refers to the identification and quantitative analysis of all metabolite components in a particular tissue or cell of an organism [21]. At present, metabolite composition analysis is mainly achieved by liquid chromatography–mass spectrometry (LC-MS), gas chromatography–mass spectrometry (GC-MS), nuclear magnetic resonance (NMR), Fourier transform infrared spectroscopy (FTIR), capillary electrophoresis–mass spectrometry (CE-MS), LC-MS/MS (liquid chromatography–tandem mass spectrometry) and so on [22,23]. The rapidly developing and widely-used LC-MS/MS technology can meet the requirements of general quantitative analysis accuracy, with good reproducibility and linear range [24]. LC-MS/MS can help find the changes of different metabolites and metabolic characteristics in the fungi–bacteria co-culture system to infer the fungi–bacteria interaction and its influence on each other’s growth and lignin degradation process.

In this research, the metabolic profiles of fungi and bacteria were analyzed using metabolomics technology based on LC-MS/MS. The fungi–bacteria interaction in the co-culture system and the effects of interaction on the microbial growth and lignin degradation process were analyzed by variation in metabolic properties and screening of differential variables. The key interaction substances were verified by the complementation experiments. This research can elucidate the mutualism mechanism of lignin degradation by microbial consortium and clarify the exchange of substances between fungi and bacteria.

## 2. Results and Discussion

### 2.1. Metabolomics Analysis of Different Lignin Degradation Systems

Metabolomics studies are expected to obtain data on the abundance of large amounts of metabolites in biological degradation systems, and the abundance contains information about the biological phenomenon under research, which is manifested in (co-) variations in the abundance of metabolites [25]. In this research, three groups containing fungal samples (labeled F), bacterial samples (labeled B), and original J-6 samples (labeled 6) were set up for comparison, and metabolomics analysis was carried out. To ensure the output of high-quality data, quality control of the assay process was performed. The TIC plots of the experimentally detected samples (Figure S1) showed good sample quality, good experimental methodology, and good system stability. Through the analysis of the above data, the key interaction substances produced in the process of microbial degradation of lignin were speculated, and the fungi–bacteria interaction mechanisms were analyzed.

#### 2.1.1. Metabolite Classification Statistics

The kinds and proportions of metabolites found in the samples were determined by statistical analysis, with the result displayed in Table 1. The metabolites are mainly composed of organoheterocyclic compounds (31.469%), organic acids and derivatives (24.126%), lipids and lipid-like molecules (14.336%) and benzenoids (8.042%). Similarly, Zhu et al. [26] analyzed the mechanism of lignin degradation by Aspergillus fumigatus G-13 and obtained that the types of differential metabolites were mainly lipids and lipid-like molecules, organic acids and their derivatives, and organoheterocyclic compounds. The results of this study were consistent with their research. The result also indicated that lignin might be depolymerized by extracellular oxidative enzymes into simple monomeric forms such as benzene ring-type compounds such as coumarin alcohol, coniferyl alcohol, and sinapyl alcohol [27], which were subsequently metabolized by various intracellular enzymes into the intermediates, such as syringic acid, vanillic acid, and p-hydroxybenzoic acid [28].

#### 2.1.2. Principal Component Analysis (PCA)

**Figure 1:**
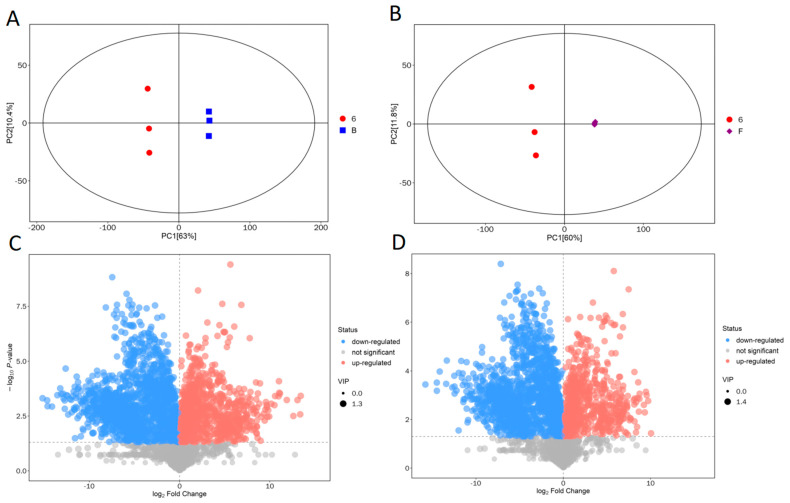
Basic data analysis of metabolomics: (**A**) Score scatter plot of PCA model for J-6 consortium vs. J-6 bacterial consortium; (**B**) score scatter plot of PCA model for J-6 consortium vs. J-6 fungal consortium; (**C**) volcano plot for J-6 consortium vs. J-6 bacterial consortium; (**D**) volcano plot for J-6 consortium vs. J-6 fungal consortium.

The first and second principal component scores were shown by the horizontal coordinate PC1 and the vertical coordinate PC2, respectively, in the scatter plots of PCA scores (Figure 1A,B). The more similar the types and contents of metabolites in the samples were, the closer the distribution of the sample points [29]. The result showed that the degree of dispersion of the sample points was higher in the fungi–bacteria consortium than that in the single fungal consortium or single bacterial consortium sample points. This clearly indicated that the degree of difference in metabolic levels was overall greater in the fungi–bacteria mixed consortium compared to the single fungal consortium or single bacterial consortium.

#### 2.1.3. Screening of Differential Metabolites

The volcano plots were created by combining the findings of the univariate and multivariate statistical analyses to test for different metabolites (Figure 1C,D). Every point on the volcano plot represented a peak. The vertical coordinate indicated the *p*-value of the Student’s *t*-test (derived from the negative of the logarithm of base 10) and the horizontal coordinate indicated the fold change of the group comparing each substance. The scatter size corresponded to the value of the variable importance projection (VIP) in the OPLS-DA model, where a larger scatter indicated a higher VIP value. Red points represented significantly up-regulated metabolites, blue points represented significantly down-regulated metabolites, and gray points represented metabolites that were not significantly different [30]. The volcano plot results showed that there were many types of differential metabolites in different metabolic systems, and further analysis of the key substances was needed.

**Figure 2:**
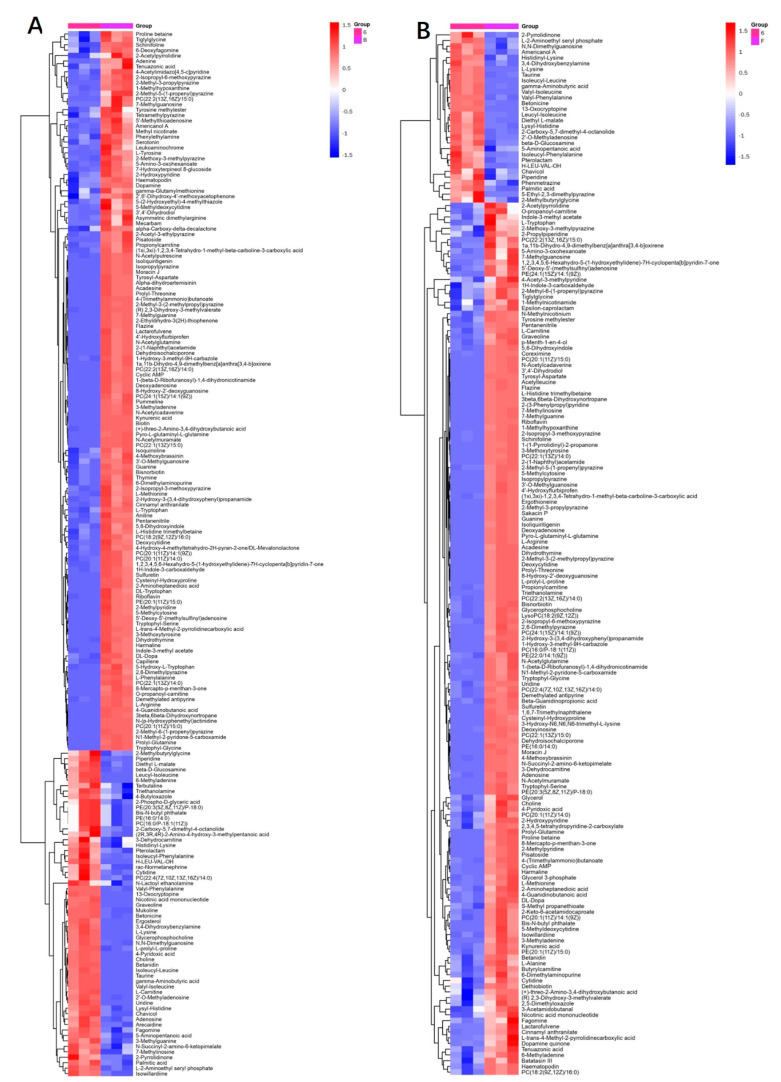
Heatmap of hierarchical clustering analysis: (**A**) J-6 consortium vs. J-6 bacterial consortium; (**B**) J-6 consortium vs. J-6 fungal consortium.

In addition, hierarchical clustering analysis (HCA) was performed in order to discover the characteristics of metabolite variation among experimental groups (Figure 2). The highly expressed substances in each group illustrated in the clustered heatmaps include the substances in Table 2, thus suggesting that these substances may be biologically similar/complementary in outcome and function and may be involved together in the same metabolic processes.

#### 2.1.4. Matchstick Analysis of Differential Metabolites

**Figure 3:**
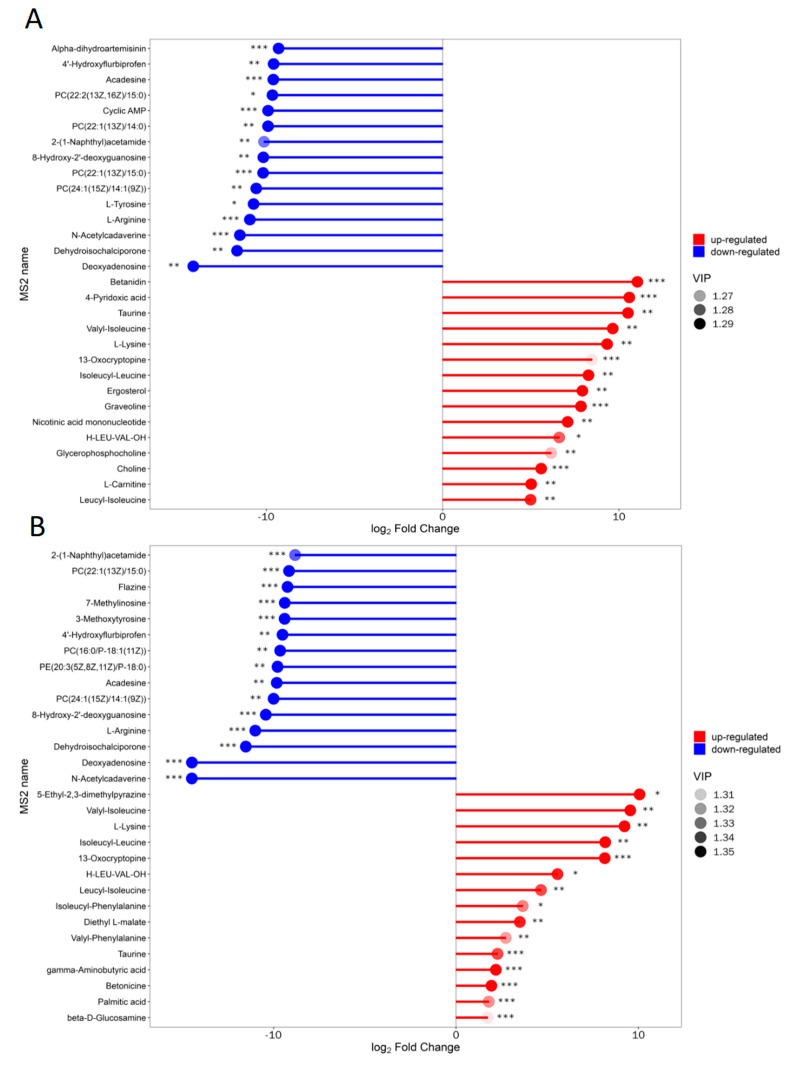
Matchstick analysis of different lignin degradation systems: (**A**) J-6 consortium vs. J-6 bacterial consortium; (**B**) J-6 consortium vs. J-6 fungal consortium. * stands for significance: * 0.01 < *p* < 0.05, ** 0.001 < *p* < 0.01, *** *p* < 0.001.

The matchstick plots (Figure 3) demonstrated differential metabolites with a large degree of change. The expression of corresponding enzyme genes might be strongly activated or strongly inhibited (* represents significance). The horizontal axis of the figure represented the change multiple after logarithmic conversion, while the depth of the dot color indicated the VIP value [31]. This analysis showed that the substances that were significantly up-regulated in the mixed system compared to the bacterial system were betanidin, 4-pyridoxic acid, 13-oxocryptopine, graveoline, choline, and so on. The substances that were significantly up-regulated in the mixed system compared to the fungal system were 13-oxocryptopine, taurine, gamma-aminobutyric acid, betonicine, palmitic acid, beta-D-glucosamine, and so on.

#### 2.1.5. KEGG (Kyoto Encyclopedia of Genes and Genomes) Annotation and Enrichment Analysis of Differential Metabolites

KEGG pathway map contains reaction/interaction networks for species metabolism, genetic information processing, and so on. It not only provides all possible metabolic pathways for the interconversion of biochemicals but also contains comprehensive annotations of the enzymes that catalyze each step [32]. All pathways mapped by the corresponding species differential metabolites were collated, differential metabolites were labeled on KEGG pathway maps, and information such as the flow of up-/down-regulated significant differential metabolites and their metabolic responses was also collated.

**Figure 4:**
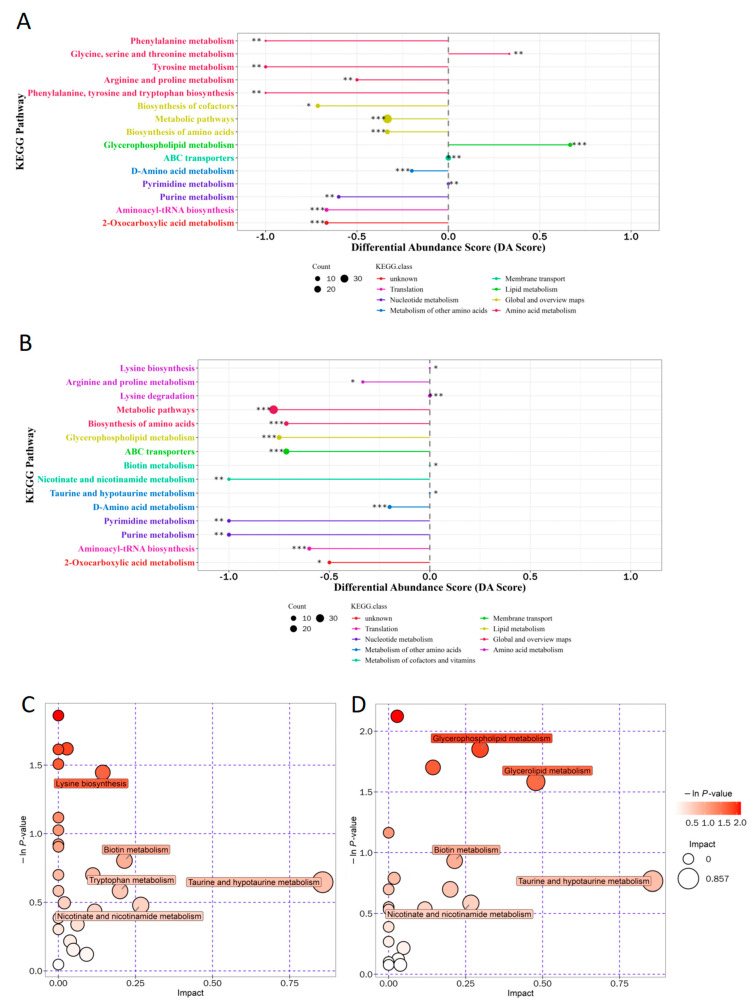
Functional annotation analysis of metabolomics: (**A**) differential abundance score for J-6 consortium vs. J-6 bacterial consortium; (**B**) differential abundance score for J-6 consortium vs. J-6 fungal consortium; (**C**) metabolic pathway analysis bubble plot for J-6 consortium vs. J-6 bacterial consortium; (**D**) metabolic pathway analysis bubble plot for J-6 consortium vs. J-6 fungal consortium. * stands for significance: * 0.01 < *p* < 0.05, ** 0.001 < *p* < 0.01, *** *p* < 0.001.

The enrichment results of differential metabolites in the KEGG metabolic pathway were presented in differential abundance score graphs (Figure 4A,B). The DA Score (differential abundance score) of the graphs reflected the overall change of all metabolites in the metabolic pathway. Therein, a score of 1 indicated that the expression trend of all annotated differential metabolites in the pathway was up-regulated, and −1 indicated that the expression trend of all annotated differential metabolites in the pathway was down-regulated. The length of the line segment indicated the absolute value of the DA Score. The size of the dots indicated the number of annotated differential metabolites in the pathway, and a larger dot indicated a higher number of differential metabolites in the pathway [33]. The results showed that the fungi–bacteria mixed system compared to the bacterial system in membrane transport—glycerophospholipid metabolism, ABC transporter, nucleotide metabolism—pyrimidine metabolism, amino acid metabolism—glycine, serine, and threonine metabolism, the overall expression of metabolites tended to be up-regulated. Meanwhile, the overall expression of metabolites in amino acid metabolism—lysine biosynthesis, lysine degradation, metabolism of taurine and hypotaurine, metabolism of cofactors and vitamins—biotin metabolism tended to be up-regulated in the fungi–bacteria mixed system compared to the fungal system.

#### 2.1.6. Metabolic Pathway Analysis of Differential Metabolites

The results of the metabolic pathway analysis were presented in bubble plots, represented in Figure 4C,D. Each bubble in the plot corresponded to a specific metabolic pathway. The horizontal position and size of the bubble indicated the impact factor of the pathway in the topology analysis, where larger bubbles meant higher impact factors. The vertical position and color of the bubble reflected the *p*-value of the enrichment analysis, where the color intensity represented the significance level, and darker-colored bubbles indicated smaller *p*-values, indicating a higher degree of enrichment significance [34]. Large dark bubbles (the pathways with higher enrichment) were selected as the focus of subsequent research. The comparison between the fungi–bacteria mixed system and the bacterial system showed differences in lysine biosynthesis, biotin metabolism, tryptophan metabolism, taurine, and hypotaurine metabolism, as well as nicotinate and nicotinamide metabolism. On the other hand, the comparison between the fungi–bacteria mixed system and the fungal system revealed distinctions in glycerophospholipid metabolism, glycerolipid metabolism, biotin metabolism, tryptophan metabolism, taurine, and hypotaurine metabolism, as well as nicotinate and nicotinamide metabolism.

#### 2.1.7. Analysis of Interacting Substances

The fungal versus bacterial system and the bacterial versus fungal system were analyzed using the same methods as previously described. Based on the inference from the literature that utilizes similar methodologies to investigate the interaction of substances [16], it is likely that substances that are significantly up-regulated in both the fungi–bacteria mixed system versus the bacterial system and the fungal system versus the bacterial system are released by the fungi. Conversely, substances that are significantly up-regulated in both the fungi–bacteria mixed system versus the fungal system and the bacterial system versus the fungal system are likely released by the bacteria. And these substances may be the most important interacting substances between fungi and bacteria.

In order to screen for more accurate interacting substances, the differential metabolites obtained by the above metabolomics analysis were screened with FOLD_CHANGE (up-regulation value) greater than 2 (those lower than 2 were not screened), and at the same time satisfied the *p*-value value of less than 0.05. As shown in Table 2, the substances that were significantly up-regulated in the fungi–bacteria mixed system compared with the bacterial system and the fungal system compared with the bacterial system were betanidin, 4-pyridoxic acid, graveoline, ergosterol, nicotinic acid mononucleotide, glycerophosphocholine, and so on. The substances that were significantly up-regulated in the mixed system versus the fungal system and the bacterial system versus the fungal system were mainly L-phenylalanine and taurine.

In summary, through synthesizing the metabolic levels of each metabolite and their roles in the pathway, it could be hypothesized that the key compounds released by the fungi that might affect the bacterial system were ergosterol, betanidin, glycerophosphocholine, 4-pyridoxic acid, gravoline, and nicotinic acid mononucleotide. The key compounds released by the bacteria that might affect the fungal system were L-phenylalanine and taurine. Among these substances, betanidin and glycerophosphocholine may play a role in regulating osmosis in microbial cells. Betanidin serves as the synthetic precursor of betalain. Betalain can be involved in osmoregulatory processes that help sustain normal physiological functions. Additionally, it can act as a non-enzymatic antioxidant, aiding in the neutralization of excessive reactive oxygen species during unfavorable conditions to maintain standard cellular metabolic activities [35]. Glycerophosphocholine, recognized as a vital component of cell membrane structure, exhibits a dose-dependent elevation in cellular glycerophosphocholine levels in response to high osmolarity [36]. Glycerophosphocholine has been identified as the preferred osmoprotectant over other endogenous metabolites [37]. Nicotinic acid or its derivatives of nucleosides and mononucleotides are important intermediates in the synthesis of a variety of body-required bioproducts, thus, nicotinic acid mononucleotide may be associated with microbial energy-producing metabolism. Ergosterol and taurine may be associated with microbial stress adaptation and may enhance microbial resistance. Higher levels of ergosterol are linked to improved resistance against low temperature, freezing, low sugar, alcohol, and oxidative stress in the yeast. Meanwhile, the yeast with decreased ergosterol content exhibited reduced adaptation to high osmotic stress [38]. Chen et al. [39] found that taurine has an effect on regulating cellular metabolism and enhancement of stress resistance. Previously, several studies have demonstrated that phenylalanine affects metabolism through induction under stress conditions [40,41]. Therefore, L-phenylalanine may affect microbial metabolism through induction.

### 2.2. Verification of Key Interacting Substances

The effects of these key compounds on lignin degradation and microbial growth were experimentally verified. The compounds released by the fungi and added to the bacterial culture system were ergosterol, betanidin, nicotinic acid mononucleotide, 4-pyridoxalic acid, gravoline, and glycerophosphorylcholine. The compounds released by the bacteria and added to the fungal culture system were L-phenylalanine and taurine, respectively.

**Figure 5:**
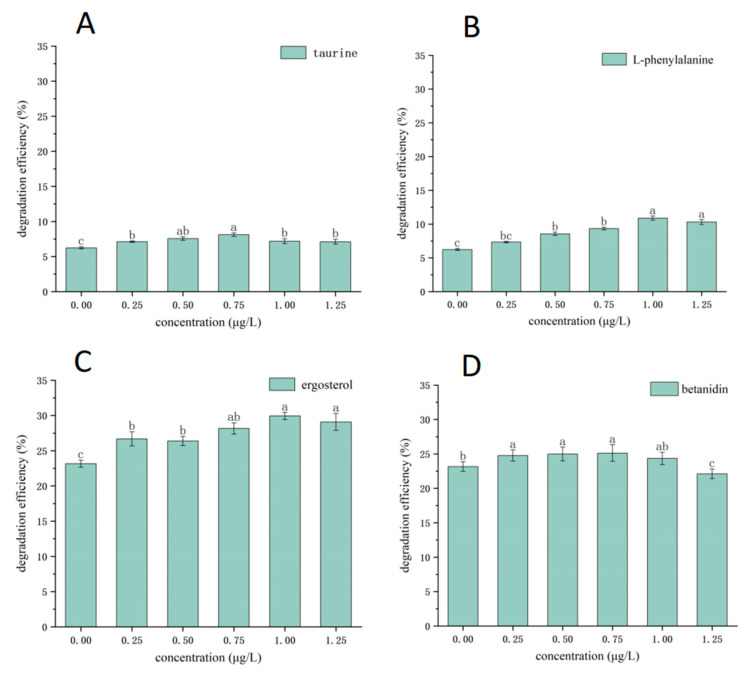
Verification of key interacting substances in the fungi–bacteria lignin degradation system: (**A**): lignin degradation efficiency of fungal culture systems adding taurine; (**B**) lignin degradation efficiency of fungal culture systems adding L-phenylalanine; (**C**) lignin degradation efficiency of bacterial culture systems adding ergosterol; (**D**) lignin degradation efficiency of bacterial culture systems adding betanidin). Different letters in the graph indicate significant differences (*p* < 0.05).

**Figure 6:**
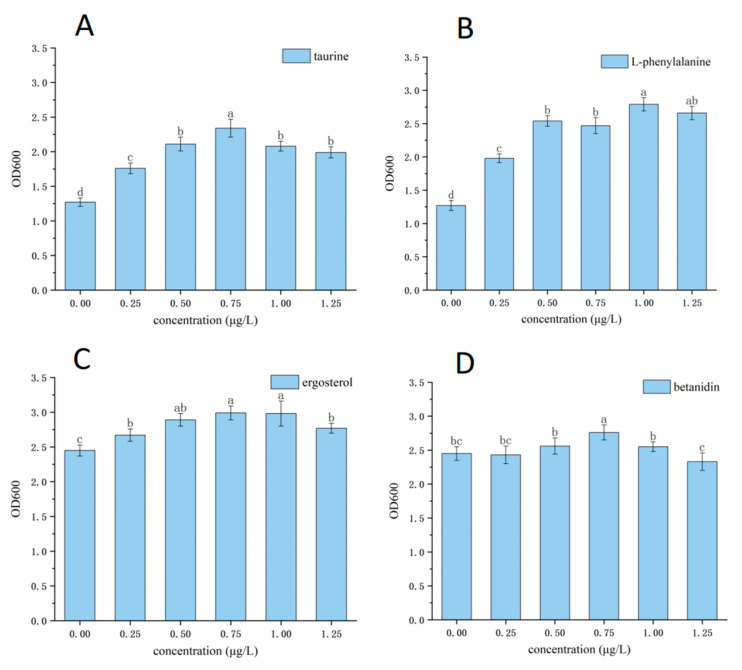
Verification of key interacting substances in the fungi–bacteria lignin degradation system: (**A**) biomass of fungal culture systems adding taurine; (**B**) biomass of fungal culture systems adding L-phenylalanine; (**C**) biomass of bacterial culture systems adding ergosterol; (**D**) biomass of bacterial culture systems adding betanidin). Different letters in the graph indicate significant differences (*p* < 0.05).

It could be seen from Figure 5 that in the experiment using alkali lignin as a degradation substrate within a certain concentration range, four interacting substances, i.e., taurine, L-phenylalanine, ergosterol, and betanidin, significantly promoted the degradation of lignin. The increase in fungi–bacteria interacting substances concentration correlated with an increase in the degradation efficiency of lignin. The lignin degradation efficiency reached the highest value when the concentration was 0.75–1 μg/L, and the degradation efficiency was 8.11% (taurine, 0.75 μg/L), 10.89% (L-phenylalanine, 1 μg/L), 29.94% (ergosterol, 1 μg/L), 25.12% (betanidin, 0.75 μg/L), respectively. Compared with the blank experimental group (bacterial consortium: 23.16%; fungal consortium: 6.24%), the degradation efficiency was improved. From Figure 6, it can be seen that when the lignin degradation efficiency was higher, the growth of the microorganisms was also better. In addition, the effects of these compounds and their analogs on lignin degradation systems have also been reported. For example, the research of Wang et al. [42] proved that ergosterol was a kind of ligninolytic-inducing compound in the incubation of *Phanerochaete sordida* YK-624 and they also identified and synthesized the ergosterol metabolites ergosta-4,7,22-triene-3,6-dione and ergosta-4,6,8(14),22-tetraen-3-one, which were compounds that also enhanced lignin-degrading activity. However, the other four interacting substances (4-pyridoxic acid, gravorin, choline glycerophosphate, and niacin mononucleotides) had no significant effect on the degradation of lignin. Compared with the experiments we did before, under the same conditions (optimal lignin degradation conditions), the J-6 microbial consortium can degrade lignin up to 54% efficiently [43]. In contrast, in this experiment, the J-6 bacterial consortium reached a degradation rate of 29.94% in the presence of ergosterol from fungi, and this degradation rate was the highest degradation rate among all groups in this experiment. It was hypothesized that fungi and bacteria in the microbial consortium may degrade lignin more efficiently by releasing interacting metabolites compared to the fungal consortium or bacterial consortium alone.

In the experiment with lignocellulose biomass as the substrate, the degradation efficiency of lignin did not change significantly with the increase in the concentration of fungi–bacteria interacting substances. It was hypothesized that the fungi or bacteria consortium alone might not be adapted to an environment with biomass as a substrate but can be adapted to an environment with alkaline lignin as a substrate. Therefore, when using biomass as a substrate, it was more necessary to use the fungi–bacteria mixed system for lignin degradation. This has also been confirmed in our previous research [11].

### 2.3. Comparison of Lignin Degradation Products

To further validate the roles of these key interacting substances in the degradation of lignin by microbial consortium, additional experiments were conducted. The lignin degradation products produced by a bacterial/fungal culture system incorporating ergosterol and L-phenylalanine, which significantly increased the efficiency of lignin degradation, were analyzed. Additionally, the lignin degradation products from a bacterial/fungal culture system where these two substances were not added were also analyzed. The results (Table 3) showed that different microbial consortia have different characteristics in the type of degradation products. For example, most of the degradation products of bacterial consortium are substances with high molecular weights, such as 13-oxocryptopine, and substances containing multiple benzene rings, such as 5,5′-dimethoxy-3,3′,7,7′-tetramethyl-2,2′-binaphthalene-1,1′,4,4′-tetrone. However, with the addition of ergosterol, lignin degradation products of high molecular weight or containing multiple benzene rings are significantly reduced. In contrast, the degradation products of fungal consortium hardly contain compounds with multiple benzene rings but contain many metabolic intermediates such as L-alanyl-L-glutamine, L-citrulline, as well as many small molecules, such as 1,7-diphospho-1-epi-valienol and selenohomocystine. In addition, with the addition of L-phenylalanine, small molecule lignin degradation products increased, and there was not much change in metabolite types in general.

Due to the limited detection time of GC-MS analysis, not all lignin degradation products were detected, but the major products of lignin degradation were detected. For example, Zhang et al. [43] used GC-MS analysis to detect lignin degradation products such as phenol, benzaldehyde, 3-methyl, butylated hydroxytoluene, benzenebutyric acid,2,3-dimethoxy, etc., were also detected in this experiment.

### 2.4. The Fungi–Bacteria Interaction Mechanism of Microbial Consortium

Through the metabolomics analysis of various data, eight key fungi–bacteria interacting substances, including taurine, L-phenylalanine, ergosterol, betanidin, 4-pyridoxic acid, gravoline, glycerophosphorylcholine, and nicotinic acid mononucleotide were screened. Through experimental verification, taurine, L-phenylalanine, ergosterol, and betanidin were found to most significantly enhance the efficiency of lignin degradation by a single consortium, especially the J-6 bacterial consortium, which achieved a 29.94% degradation rate with the addition of ergosterol, a 29.27% increase compared to the bacterial consortium without ergosterol. Moreover, as the efficiency of lignin degradation increased, so did the biomass. Subsequent analysis of degradation products from microbial consortia with and without the addition of these key interactive substances showed that with the addition of ergosterol, high molecular weight or polycyclic aromatic metabolites in the bacterial consortium significantly decreased. With the addition of L-phenylalanine, intermediate metabolites in the fungal consortium decreased while small molecule metabolites increased. This indicated that these two key interactive compounds played a significant role in achieving complete degradation of lignin.

**Figure 7:**
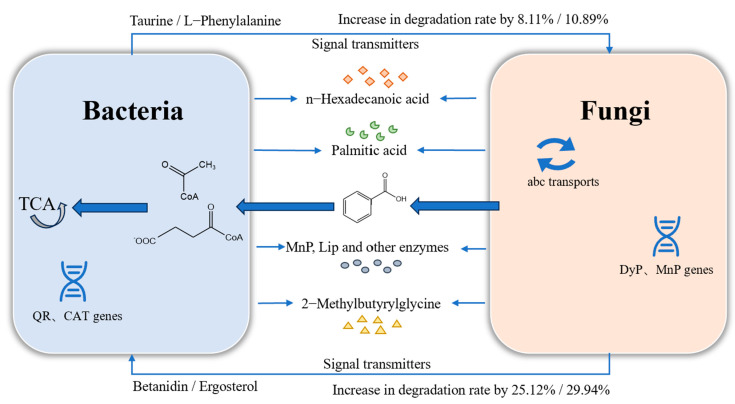
Degradation mechanism of lignin consortium system and key interacting substances interaction mechanism.

Based on the above analysis, a hypothesis regarding the interaction mechanism of these key chemical currencies in the degradation of the lignin mixed system was formulated (Figure 7). Betanidin from fungi may play a role in regulating the osmotic pressure of bacterial cell membranes, while ergosterol could be involved in stress-adaptive regulation and enhancing stress resistance in bacteria. Additionally, L-phenylalanine from bacteria may be involved in inducing cellular metabolism in fungi, while taurine could play a role in stress-adaptive regulation and enhancing stress resistance in fungi.

## 3. Materials and Methods

### 3.1. Materials

The microbial consortium J-6 was obtained from our previous research and stored at 4 °C in a refrigerator [43]. The fungi of J-6 were dominated by Saccharomycetales (98.92%) and the bacteria were mainly *Cupriavidus* sp. (29.84%), *Shinella* sp. (47.38%), and *Bosea* sp. (7.96%). This experiment was conducted using common lignocellulosic biomass in China, including bagasse (Guangxi, China), eucalyptus root (Guangxi, China), and corn straw (Heilongjiang, China). The lignin content of the biomass was from 16.37% to 26.31% [11]. In addition, the main experimental reagents, such as alkaline lignin, betanidin, ergosterol, taurine, L-phenylalanine, magnesium sulfate, and so on, were purchased from Shanghai Aladdin Biochemical Technology Co., Ltd. (Shanghai, China). The reagents were analytically pure.

### 3.2. Culture Medium

#### 3.2.1. Basic Medium

The basic medium consisted of the following components: 2.5 g/L sodium chloride, 2.5 g/L (NH_4_)_2_SO_4_, 1 g/L potassium dihydrogen phosphate, 0.2 g/L MgSO_4_·7H_2_O, 0.1 g/L calcium chloride, 0.5 g/L glucose, 1 mL/L trace elements and 0.5 g/L alkaline lignin, which were acquired from our prior experiments [43]. Additionally, 1 mol/L NaOH or HCl solution was added to the mixture in order to regulate the pH level.

#### 3.2.2. Degradation Medium

The composition of the degradation medium was as follows: biomass (1.6 g/L) or alkaline lignin (0.5 g/L), 0.2 g/L MgSO_4_, 1.0 g/L KH_2_PO_4_, 2.5 g/L NaCl, 2.5 g/L (NH_4_)_2_SO_4_, 0.1 g/L CaCl_2_, 0.5 g/L glucose, and 1 mL/L trace elements. Trace elements solution formulation was as follows: 0.1 g/L ZnSO_4_·7H_2_O, 0.16 g/L CoCl_2_·6H_2_O, 0.15 g/L CuSO_4_·5H_2_O, 0.1 g/L MnSO_4_·H_2_O, 0.02 g/L H_3_BO_3_, 0.8g/L Na_2_MoO_4_·8H_2_O, and 0.05 g/L NiCl_2_·6H_2_O.

### 3.3. Isolation of Fungal and Bacterial Consortium

According to the available study [44], the isolation of fungal bacteria was done by the use of antifungal agents and antibiotics, and this isolation method was proven to be effective. This method was also applied in this study.

For bacterial isolation, the J-6 consortium suspension was diluted in 10 times the volume of sterile saline, and the diluted consortium suspension was shaken in a vortex apparatus for homogenization. The consortium suspension was moved to the basic medium with a 10% inoculum volume. At the same time, 0.05 g/L of nystatin and 0.2 g/L of cycloheximide were added to inhibit the growth of fungi. After that, it was put into a constant-temperature shaker to be cultured for 24 h at 37 °C and 200 rpm.

When the fungi were isolated, the suspension was inoculated into the culture medium according to the above method. Then, 0.1 g/L penicillin and 0.1 g/L streptomycin were added to inhibit the growth of bacteria before incubation, followed by being put into the constant-temperature shaker at 28 °C and 200 rpm for 3–7 d after the inoculation was completed.

The above isolated bacterial and fungal consortia were transformed and amplified three times, respectively. Then the colony composition was analyzed to ensure complete isolation by the sequencing method. After analyzing the community structure, it was determined that the fungi and bacteria had been completely isolated.

### 3.4. Metabolomics Analysis

#### 3.4.1. Analytics Information and Raw Data Preprocessing

Due to the complexity of natural lignin, the use of stable lignin purification products is an effective and conventional method to study the microbial degradation behavior and mechanism of lignin, and there is great similarity between the two structures [45]. Therefore, in the process of metabolomics research in this study, the carbon source used was alkali lignin.

Three groups of control experiments were conducted separately, inoculating J-6 consortium (labeled 6), J-6 fungal consortium (labeled F), and J-6 bacterial consortium (labeled B) into the alkaline lignin degradation medium, and lignin degradation was conducted in a constant-temperature shaker at 30 °C and 200 rpm for 48 h to obtain the test samples. Then, 100 μ L of different samples were transferred to an EP tube (Eppendorf tube). After the addition of 400 μ L of extract solution (acetonitrile:methanol = 1:1), the samples were vortexed for 30 s, sonicated for 10 min in and ice-water bath, and incubated for 1 h at −40 °C. Then, the samples were centrifuged at 13,800× *g* for 15 min at 4 °C. The resulting supernatant was transferred to a fresh glass vial for LC-MS/MS analysis. Specifically, the ionization source of the Orbitrap platform was electrospray ionization. The positive and negative ion modes were combined to detect the metabolome.

This analysis consisted of four stages, which were basic data analysis, differential metabolite screening, advanced data analysis, and speculation of interacting substances. The original data was prepared and sorted through deviation filtering, missing value filtering, missing value filling, and data standardization.

#### 3.4.2. Basic Data Analysis

Hierarchical clustering analysis (HCA) was performed on all samples and their metabolites, and the substances with high expression were screened. Metabolite classification statistics were carried out to classify the identified metabolites according to the chemical classification information. The metabolite classification and proportion pie chart were drawn, focusing on the types of metabolites that accounted for a relatively large proportion. Multivariate analysis (MVA) was conducted with principal component analysis (PCA), orthogonal partial least squares-discriminant analysis (OPLS-DA) and so on. Through PCA using SIMCA software (V16.0.2, Sartorius Stedim Data Analytics AB, Umea, Sweden), the overall distribution trend as well as the degree of difference of samples between groups, were obtained. To gather more accurate data regarding the differences between metabolite groups and the level of correlation of the experimental group, non-orthogonal and orthogonal variables were analyzed independently after the orthogonal variables in the metabolites that were not correlated with the categorical variables were filtered out using OPLS-DA in SIMCA software. Univariate analysis (UVA) was studied with a Student’s *t*-test and analysis of variance (ANOVA) to obtain independent changes in metabolite levels.

#### 3.4.3. Differential Metabolite Screening

Combining the results of univariate and multivariate statistical analyses, the differential metabolites were screened, and volcano plots were used to illustrate the findings of screening differential metabolites, which intuitively demonstrated the overall distribution of the metabolite differences between groups.

#### 3.4.4. Advanced Data Analysis

Matchstick plot analysis of differential metabolites was carried out to compare each group, calculate the ratio corresponding to the quantitative value of differential metabolites, and screen out the different metabolites with greater variation through the matchstick diagram analysis. KEGG annotation of differential metabolites was to sort out various relevant information about pathways in the KEGG Pathway database and draw pathway maps. According to the enrichment results of differential metabolites in KEGG metabolic pathways, the general changes of all differential metabolites in a pathway were obtained. Besides, the pathways whose overall expression tended to be up-regulated were screened out. Metabolic pathway analysis of differential metabolites was performed to find out the target pathway where differential metabolites had a greater impact on the pathway through the comprehensive analysis of the pathway where differential metabolites reside (including enrichment analysis and topological analysis).

#### 3.4.5. Speculation on Fungi–Bacteria Interacting Substances

The key fungi–bacteria interacting substances were analyzed by combining the metabolic levels of these metabolites and their roles and effects in the pathway.

### 3.5. Complementation Experiments with Interacting Substances

The isolated bacterial/fungal suspensions were inoculated into the medium containing the interaction compounds to be verified at a 10% inoculation rate, and the concentration gradients of the interaction compounds to be verified were set as 0 μg/L, 0.25 μg/L, 0.5 μg/L, 0.75 μg/L, 1 μg/L, and 1.25 μg/L, respectively. Three parallel experiments were set for each concentration gradient. After inoculation, the samples were incubated at 30 °C and 200 rpm in a constant-temperature shaker. Throughout the incubation period, samples were sampled periodically. The incubation time of the biomass degradation system was 14 d, and the incubation time of the alkaline lignin degradation system was 48 h (the incubation conditions and incubation time were determined according to the previous experiments).

### 3.6. Analytical Methods

LC-MS/MS analyses were performed using a UHPLC system (Vanquish, Thermo Fisher Scientific, Waltham, MA, USA) with a UPLC BEH Amide column (2.1 mm × 100 mm, 1.7 μm) coupled to Q Exactive HFX mass spectrometer (Orbitrap MS, Thermo Fisher Scientific, USA). The mobile phase consisted of 25 mmol/L ammonium acetate and 25 mmol/L ammonia hydroxide in water (pH = 9.75) (A) and acetonitrile (B). The auto-sampler temperature was 4 °C, and the injection volume was 2 μL.

According to the previously reported method, soluble lignin concentration was measured by absorbance at 280 nm [46]. The NREL (National Renewable Energy Laboratory) method of the U.S. Department of Energy was used to determine the lignin content in biomass [47]. The absorbance value of the microorganism suspension at 600 nm was measured using a visible spectrophotometer (TU-1901, Purkinje General Instrument, Beijing, China). The community structure analysis method was high-throughput sequencing [43].

The lignin degradation products were analyzed by gas chromatography–mass spectrometry (GC-MS) analysis, which was performed using previously reported methods [48].

### 3.7. Statistical Methods

The above experiments were replicated three times, and the average data were recorded. One-way ANOVA was employed to identify any significant bias in the results (Tukey test, *p* < 0.05).

## 4. Conclusions

Investigating the fungi–bacteria interaction mechanism during lignin degradation to improve the degradation efficiency of lignin is of great significance for global carbon cycling and value-added utilization of biological resources research. The results showed that the positive interaction between fungi and bacteria could improve the efficiency of lignin degradation. The metabolites released by fungi, especially betanidin and ergosterol, had an impact on bacterial metabolism, promoted the degradation of macromolecules, and significantly increased the lignin degradation efficiency. Metabolites released by bacteria, especially L-phenylalanine and taurine, played a key role in fungal metabolism, leading to more complete degradation. The interaction mechanism of chemical currencies exchange between fungi and bacteria during lignin degradation obtained in this study can provide theoretical guidance for microbial consortium degradation technology. In future research, it is necessary to utilize the results of this study to develop suitable biodegradation enhancement methods for different lignin degradation systems in order to optimize the lignin degradation process.

## Figures and Tables

**Table 1:** Metabolite classification and proportion for all samples (including the J-6 consortium and J-6 bacterial/fungal consortium).

Super Class	Percentage
Organoheterocyclic compounds	31.47
Organic acids and derivatives	24.13
Lipids and lipid-like molecules	14.34
Benzenoids	8.04
Organic oxygen compounds	7.69
Nucleosides, nucleotides, and analogues	4.55
Organic nitrogen compounds	3.85
Phenylpropanoids and polyketides	3.15
Alkaloids and derivatives	2.10
Organic 1,3-dipolar compounds	0.35

**Table 2:** Major differential metabolites in lignin degradation systems.

J-6 and J-6 Bacterial Consortium			J-6 and J-6 Fungal Consortium		
Name	Fold-Change	*p*-Value	Name	Fold-Change	*p*-Value
Betanidin	2113.76	0.00028	Taurine	4.83	0.000036
4-Pyridoxic acid	1536.90	0.00039	L-Phenylalanine	2.11	0.031
Ergosterol	243.13	0.0016			
Graveoline	229.67	0.00051			
Nicotinic acid mononucleotide	135.94	0.0019			
Glycerophosphocholine	71.14	0.0012			

**Table 3:** Identification of the products of the lignin degradation system.

No.	RT ^1^	Compound	Bacterial Consortium		Bacterial Consortium + Ergosterol		Fungal Consortium		Fungal Consortium + L-Phenylalanine	
			1d	2d	1d	2d	1d	2d	1d	2d
1	10.45 min	Phenol		+		+		+		+
2	11.58 min	Ethyl 3-hydroxybutyrate			+		+	+	+	
3	13.27 min	Benzaldehyde, 3-methyl		+	+	+	+	+	+	+
4	18.32 min	5,5′-Dimethoxy-3,3′,7,7′-tetramethyl-2,2′-binaphthalene-1,1′,4,4′-tetrone	+	+						
5	19.81 min	Butylated Hydroxytoluene				+				+
6	22.79 min	Benzenebutyric acid, 2,3-dimethoxy-	+	+	+		+	+	+	
7	23.77 min	1,2-Benzenedicarboxylic acid, butyloctyl ester	+	+	+		+		+	
8	23.77 min	Phthalic acid, hept-3-yl isobutyl ester	+	+	+		+		+	
9	24.64 min	n-Hexadecanoic acid	+	+	+	+	+	+	+	+
10	26.51 min	Octadecanoic acid		+	+	+	+	+	+	+
11	32.69 min	1-Hydroxy-2-(2,3,4,6-tetra-O-acetyl-beta-D-glucopyranosyl)-9H-xanthene-3,6,7-triyl triacetate		+	+	+		+	+	+
12	34.27 min	L-Alanyl-L-Glutamine			+		+	+		+
13	35.75 min	1,7-diphospho-1-epi-valienol						+		+
14	35.82 min	Selenohomocystine				+		+	+	+
15	36.32 min	L-Citrulline					+	+	+	
16	36.33 min	3-Methoxytyrosine		+			+			+
17	36.55 min	4-(Trimethylammonio)butanoate				+		+	+	+
18	36.80 min	Chlomethoxyfen	+		+				+	
19	38.45 min	13-Oxocryptopine	+	+	+		+		+	

^1^ RT: retention time.

## Data Availability

The data are available from the corresponding author on reasonable request.

## Supplementary Materials

The following supporting information can be downloaded at: https://www.mdpi.com/article/10.3390/molecules30030508/s1, Figure S1: TIC plot of positive ionization mode by UHPLC-QE-MS detection for (the representative sample).
