# Supplementary material for: The Fungi–Bacteria Interaction Mechanism of Microbial Consortium During Efficient Lignin Degradation Based on Metabolomics Analysis
Source: Molecules. 2025 Jan 23;30(3):508. doi: 10.3390/molecules30030508 (PMC11821196; doi:10.3390/molecules30030508)
Supplement: Supplementary file 1 [file molecules-30-00508-s001.zip › molecules-3368786-supplementary.pdf]

Supplementary materials

Figure Legends:

Figure S1 TIC plot of positive ionization mode by UHPLC-QE-MS detection (the representative sample)

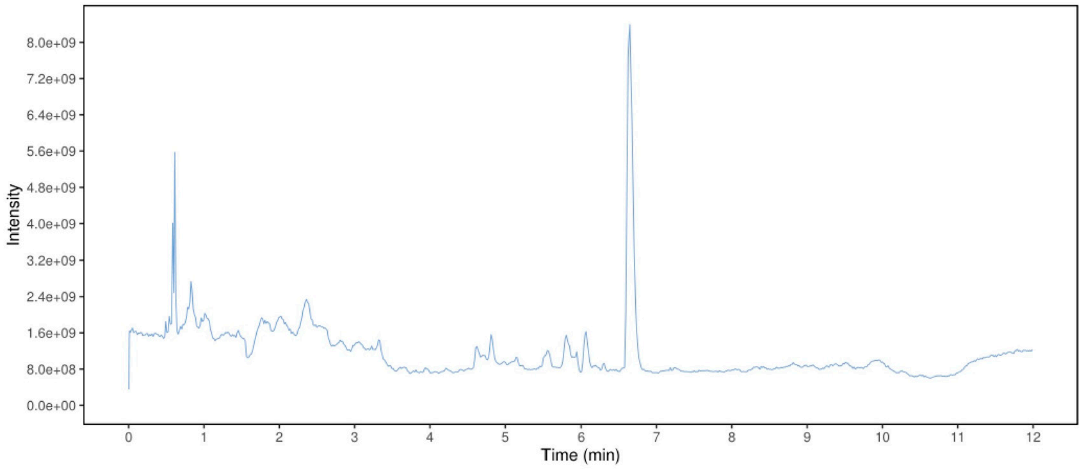

Figure S1 TIC plot of positive ionization mode by UHPLC-QE-MS detection (the representative sample)
